# Competitive phenotype and metabolite repertoire of the endophytic *Diaporthe* sp. PBS30-7

**DOI:** 10.3389/fmicb.2026.1943254

**Published:** 2026-10-15

**Authors:** Ling Yang, Chuancheng Wei

**Affiliations:** 1 Department of Pharmaceutical Life Sciences, Faculty of Pharmacy, Universiti Malaya, Kuala Lumpur, Malaysia; 2 Department of Pharmaceutical Chemistry, Faculty of Pharmacy, Universiti Malaya, Kuala Lumpur, Malaysia

**Keywords:** candidate metabolites, endophytic fungi, feature-based molecular networking, fungal competition, molecular docking

## Abstract

Endophytic fungi represent potential sources of antifungal agents, but the phenotypic and chemical basis of their interactions with competing fungi remains incompletely understood. Here, physiological characterization, time-resolved confrontation assays, cell-free culture-filtrate testing, untargeted chemical profiling, and molecular docking were integrated to characterize the competitive phenotype of the endophytic fungus *Diaporthe* sp. PBS30-7, isolated from the medicinal plant *Pereskia bleo* (Kunth) DC., and to prioritize candidate metabolites for subsequent investigation. *Diaporthe* sp. PBS30-7 exhibited condition-dependent growth and a temporally asymmetric competitive phenotype against two *Penicillium* species, characterized by sustained colony expansion, progressive restriction of target-fungal growth, and pronounced hyphal abnormalities. Cell-free culture filtrates retained antifungal activity against both target fungi, supporting a contribution of extracellular chemical factors to the overall antagonistic phenotype. Feature-based molecular networking of a culture-associated agar–mycelium extract resolved a chemically diverse repertoire that included putatively annotated beauvericin-type cyclodepsipeptides, diketopiperazines, and unresolved molecular families. Molecular docking further prioritized this candidate chemical space, with beauvericin derivatives displaying particularly favorable target-dependent docking profiles toward CYP51, Fks1, and the ABC transporter, whereas cyclo(L-Tyr-L-Tyr) emerged as a notable diketopiperazine candidate. Together, these findings prioritize candidate metabolites potentially associated with the competitive phenotype of *Diaporthe* sp. PBS30-7 and provide a foundation for future mechanistic studies of endophytic fungal antagonism.

## 1 Introduction

Fungal pathogens pose an increasing threat to human health, agricultural productivity, and global food security, while the rapid emergence of antifungal resistance continues to compromise the efficacy of existing control strategies (van Rhijn and Rhodes, 2025). Moreover, the limited chemical diversity and restricted range of cellular targets represented by current antifungal drugs highlight the need for new molecules with novel structural scaffolds (Lockhart et al., 2023). Against this backdrop, plant-associated endophytic fungi have emerged as a promising source of structurally diverse antifungal natural products.

Plant endophytic fungi comprise a taxonomically diverse assemblage of fungi that inhabit living internal plant tissues for at least part of their life cycle without causing apparent disease symptoms, although the boundaries of the endophytic lifestyle remain context dependent and continue to be debated (Liao et al., 2025; Rodriguez et al., 2009). Within host tissues, they coexist with complex microbial communities and engage in continuous ecological interactions, including competition for space, nutrients, and other limiting resources. These interactions are accompanied by substantial biosynthetic capacity and an extensive repertoire of secondary metabolites, making endophytic fungi a rich reservoir of structurally diverse bioactive compounds, including antimicrobial natural products (Liao et al., 2025; Nazir et al., 2024).

Among endophytic fungi, species of *Diaporthe* have attracted considerable attention because of their broad host range and exceptional biosynthetic potential (Chepkirui and Stadler, 2017; Wei B. et al., 2025). Members of the genus produce a wide array of secondary metabolites with antifungal and other biological activities, highlighting *Diaporthe* as an important source of bioactive natural products (Xu et al., 2021). Despite extensive investigations into the chemistry and bioactivity of *Diaporthe*, it remains unclear whether competitive interactions are reflected in cell-free extracellular antifungal activity and the composition of extracellular metabolites. Growth inhibition observed during dual-culture confrontation may arise from multiple, potentially overlapping processes, whereas metabolomic annotation alone cannot establish causal relationships between individual metabolites and the observed biological activity (Knowles et al., 2022; Nothias et al., 2018). Consequently, the relationship between competitive fungal behavior, extracellular antifungal activity, and metabolite composition remains poorly resolved. Addressing this gap requires an integrated experimental framework that combines competitive interaction assays, cell-free bioactivity, and metabolomic profiling while explicitly distinguishing biological association from mechanistic causation.

In this study, we investigated the competitive phenotype of the endophytic fungus *Diaporthe* sp. PBS30-7 against *Penicillium sumatrense*, a food-spoilage-associated mold, and *P. citrinum*, a ubiquitous environmental fungus occasionally implicated in opportunistic infections. Time-resolved dual-culture assays, microscopy, cell-free culture-filtrate testing, exploratory chemical profiling, and molecular docking were integrated to characterize fungal interactions, evaluate non-contact antifungal activity, and prioritize candidate metabolites and metabolite–target pairs.

## 2 Materials and methods

### 2.1 Fungal isolates

*Diaporthe* sp. PBS30-7 was isolated from the stem of *Pereskia bleo* (Kunth) DC. and identified by Dr. Ling Yang at the Mushroom Research Center, Universiti Malaya, based on morphological characteristics and phylogenetic analysis of the internal transcribed spacer (ITS) sequence amplified using the ITS1F and ITS4 primers, as previously described (Yang et al., 2026). The ITS sequence was deposited in GenBank under accession number PX850412, and the isolate is maintained at the Mushroom Research Center under the strain code PBS30-7. *Penicillium sumatrense* and *P. citrinum* were supplied as previously identified cultures by the Institute for Tropical Biology and Conservation, Universiti Malaysia Sabah.

### 2.2 Effects of temperature, medium, and pH on the growth of *Diaporthe* sp. PBS30-7

The growth of *Diaporthe* sp. PBS30-7 was evaluated under different temperatures, nutrients, and pH conditions using a two-stage experimental design. In the initial screening, colony growth was assessed at 23, 27, and 33 °C on potato dextrose agar (PDA; Difco™, Becton Dickinson and Company, United States), malt extract agar (MEA; Merck, Germany), and oatmeal agar (OMA). The treatment combinations are summarized in Supplementary Table 1. Based on the results of the initial screening, the culture medium supporting the highest growth rate was selected for subsequent pH experiments. Growth was then evaluated at pH 5–8 after adjustment of the medium with sterile 1 M HCl or 1 M NaOH before autoclaving (Boruta et al., 2024).

For all experiments, 5-mm mycelial plugs were excised from the actively growing margins of 7-day-old PDA cultures and centrally inoculated onto fresh agar plates. Each treatment comprised three independent biological replicates. Colony diameter was measured daily along two perpendicular axes, and the mean value was used for subsequent analyses (Fedele et al., 2024). Measurements were discontinued before colonies reached the edge of the plate to ensure comparable growth assessments among treatments. Colony pigmentation, aerial mycelial development, texture, and margin characteristics were documented after 7 days of incubation.

To standardize comparisons among treatments, a predefined endpoint (D^*^) was established as the final observation day before any replicate reached full plate coverage (90 mm). If the earliest replicate reached ≥90 mm on day t, D^*^ was defined as *t* – 1 for all treatments. When no replicate reached 90 mm during the 7-day observation period, Day 6 was used as the standardized endpoint.

### 2.3 Growth kinetic analysis

Growth kinetics were quantified by estimating the maximum specific growth rate (μ_max_) and lag phase duration (λ) using a modified Gompertz model fitted to colony diameter data by non-linear regression in GraphPad Prism v10.6.0 (GraphPad Software, San Diego, CA, United States), following previously described procedures (Boonruang and Lerkkasemsan, 2023; Passão et al., 2023; Zwietering et al., 1990). The model is expressed as:


$$\begin{array}{l} y=A+C\times exp\left(-exp\left(-B\times \left(t-M\right)\right)\right) \end{array}$$ (1)

where *y* is the colony diameter (mm) at time *t* (days), *A* is the initial colony diameter, *C* is the increase in colony diameter, *B* is the relative growth rate, and *M* is the time at which the absolute growth rate reaches its maximum.

Because colony growth was initiated from 5-mm mycelial plugs, colony diameter measurements were corrected by subtracting 5 mm before model fitting.

The maximum specific growth rate (μ_max_, cm/day) was calculated as:


$$\begin{array}{l} \mu_{\max}=\frac{\left(B\times C\right)}{e} \end{array}$$ (2)

Lag phase duration (λ, day) was calculated as:


$$\begin{array}{l} \lambda =M-\left(\frac{1}{B}\right) \end{array}$$ (3)

where *e* is the base of the natural logarithm.

### 2.4 Assessment of antagonistic activity against *Penicillium* spp.

*Diaporthe* sp. PBS30-7, *Penicillium citrinum*, and *P. sumatrense* were subcultured separately on potato dextrose agar (PDA) and incubated at 27 °C for 7 days to obtain actively growing colonies.

#### 2.4.1 Dual-culture antagonism assay

Antagonistic interactions were evaluated using dual-culture assays. A 5-mm mycelial plug of *Diaporthe* sp. PBS30-7 was placed 1 cm from the edge of a 90-mm PDA plate, while an equally sized plug of *P. citrinum* or *P. sumatrense* was positioned on the opposite side of the same plate. Each treatment comprised three independent biological replicates. Plates were sealed with Parafilm and incubated in the dark at 27 °C for 8 days. Colony expansion and interaction phenotypes were recorded throughout the incubation period. Interaction types were classified according to the morphological classification systems (Hamzah et al., 2018; Wheeler and Hocking, 1993).

#### 2.4.2 Quantification of antagonistic activity

Radial growth of *P. citrinum* and *P. sumatrense* was measured daily in dual cultures and pathogen-only controls. Measurements continued until pathogen growth had remained unchanged for at least 48 h. Antagonistic activity was expressed as the percentage of mycelial growth inhibition (MGI) (Almeida et al., 2020):


$$\begin{array}{l} MGI\left(\%\right)=\;\frac{\left(C\;-\;T\right)}{C}\;\times \;100 \end{array}$$ (4)

where *C* is the mean colony radius (mm) of the control and *T* is the mean colony radius (mm) of the dual-culture treatment.

#### 2.4.3 Microscopic analysis of antagonistic interactions

Microscopic observations were performed after 8 days of incubation to examine cellular responses at the fungal interaction zone. Agar blocks spanning the confrontation zone were aseptically excised, and mycelia were sampled from the *Diaporthe* sp. PBS30-7 region, the pathogen region, and the interaction interface. Samples were mounted in lactophenol cotton blue and examined using a compound light microscope at 400× magnification. Monocultures of each fungus were examined in parallel as controls. Hyphal alterations, including coiling, penetration, lysis, vacuolization, and abnormal conidiophore development, were documented by digital microscopy.

### 2.5 Assessment of extracellular antifungal activity

#### 2.5.1 Preparation of culture filtrates

Cell-free culture filtrates of *Diaporthe* sp. PBS30-7 were prepared using a culture-filtrate–amended medium to evaluate the contribution of diffusible extracellular metabolites to fungal antagonism (Yassin et al., 2022). Five millimeter mycelial plugs were excised from the actively growing margins of 7-day-old PDA cultures and transferred into 150-mL Erlenmeyer flasks containing 50 mL of potato dextrose broth. Flasks were incubated at 27 °C for 7 days on an orbital shaker at 150 rpm in the dark. The culture broth was filtered through sterile muslin cloth to remove mycelial biomass and subsequently centrifuged at 9,000 rpm for 15 min to remove residual spores. The clarified supernatant was sterilized by filtration through a 0.22-μm membrane filter and mixed with molten PDA at a 1:1 (v/v) ratio before being poured into sterile 90-mm Petri dishes.

#### 2.5.2 Culture filtrate bioassay

Extracellular antifungal activity was evaluated by inoculating the center of each culture-filtrate–amended PDA plate with a 5-mm mycelial plug of *Penicillium citrinum* or *P. sumatrense*. PDA supplemented with itraconazole (100 μg mL^−1^; Sigma-Aldrich, USA) served as the positive control, whereas unamended PDA served as the negative control. Each treatment comprised four independent biological replicates (*n* = 4). Plates were incubated in the dark at 27 °C for 7 days. Colony diameter was measured along two perpendicular axes, and the mean value was used to calculate the percentage of mycelial growth inhibition (MGI) according to Equation 4.

### 2.6 Preparation and chemical profiling of culture-associated metabolites

#### 2.6.1 Solid-state cultivation and extraction of the ethyl acetate-extractable metabolite fraction

*Diaporthe* sp. PBS30-7 was cultivated on potato dextrose agar (PDA) in 90-mm Petri dishes for exploratory profiling of culture-associated metabolites. Twenty plates were individually inoculated with four 5-mm mycelial plugs and incubated at 27 ± 2 °C under a 12-h light/12-h dark photoperiod for 28 days (Abramczyk et al., 2023). After incubation, the mycelium and underlying agar were cut into approximately 5-mm fragments, and the material from all 20 plates was pooled in a single extraction vessel to obtain sufficient material for chemical profiling.

Methanol extraction was performed as previously described (Yang et al., 2026). Methanol (Merck, Darmstadt, Germany) was added to the pooled agar-mycelium material at a ratio of 1 mL per gram of culture material. Extraction was performed at room temperature for 24 h on an orbital shaker at 150 rpm, after which the extract was filtered through Whatman No. 1 filter paper. The residual material was re-extracted twice under identical conditions, yielding three successive extraction cycles in total. The methanolic extracts were combined and concentrated under reduced pressure at 40 °C, and the mass of the resulting crude extract was recorded.

The crude extract was reconstituted in 20% (v/v) aqueous methanol and subjected to three successive liquid-liquid extractions with an equal volume of ethyl acetate. The ethyl acetate fractions were combined and evaporated to dryness under reduced pressure at 40 °C. The resulting ethyl acetate-extractable fraction was transferred to an amber glass vial and stored at 4 °C for 7 days before UHPLC-Orbitrap-HRMS/MS analysis (Narváez et al., 2020).

#### 2.6.2 UHPLC–Orbitrap–HRMS/MS analysis

The chromatographic conditions were adapted from previously reported LC–MS methods and refined through preliminary scouting runs (Ma et al., 2024; Mayrhofer et al., 2024; Moura et al., 2025; Ugarelli et al., 2024). The final gradient was selected to accommodate constituents spanning a broad polarity range within the ethyl acetate-extractable fraction and to elute strongly retained components within the analytical run. Before analysis, 2.00 mg of the dried ethyl acetate-extractable fraction was dissolved in LC–MS-grade methanol and adjusted to a final volume of 2.00 mL, yielding a concentration of 1.00 mg mL^−1^. The solution was passed through a 0.22-μm polytetrafluoroethylene (PTFE) syringe filter, and a 1.00-mL aliquot was transferred to an amber screw-cap autosampler vial.

The prepared fraction was analyzed using a Dionex Ultimate 3000 RSLC ultra-high-performance liquid chromatography system coupled to a Q Exactive Focus Orbitrap mass spectrometer equipped with a heated electrospray ionization source (HESI-II; Thermo Fisher Scientific, Bremen, Germany). Chromatographic separation was performed on a Waters Acquity UPLC HSS T3 column (100 × 2.1 mm, 1.8 μm) maintained at 40 °C. The mobile phases consisted of water containing 0.1% formic acid (A) and acetonitrile containing 0.1% formic acid (B) (Salis et al., 2024). The gradient program was as follows: 5% B from 0 to 5 min, a linear increase from 5% to 95% B from 5 to 42 min, 95% B from 42 to 47 min, and re-equilibration at 5% B from 47.1 to 50 min. The flow rate was 0.3 mL min^−1^, and the injection volume was 5 μL.

Mass spectrometric data were acquired in positive- and negative-ion modes within a single analytical run using polarity switching. The HESI source was operated at spray voltages of 3.0 kV in positive-ion mode and 2.5 kV in negative-ion mode. The capillary and probe-heater temperatures were set to 320 and 350 °C, respectively. The sheath-, auxiliary-, and sweep-gas flow rates were set to 35, 10, and 0 arbitrary units, respectively, and the S-lens RF level was set to 60. Full-scan mass spectra were acquired in profile mode over an m/z range of 100–1,500 at a resolution of 70,000 at m/z 200. Data-dependent MS/MS acquisition was performed using a Top-3 method at a resolution of 17,500 at m/z 200, with an isolation window of 1.5 m/z and stepped normalized collision energies of 20, 40, and 60. The automatic gain-control targets were set to 1 × 106 for full-scan MS and 2 × 105 for MS/MS, with corresponding maximum injection times of 100 and 50 ms, respectively. The minimum AGC target and precursor-intensity threshold were set to 8 × 10^3^ and 1.6 × 105, respectively (Baciero-Hernández et al., 2024). The MSX count was set to 1, and a dynamic-exclusion duration of 5 s was applied to minimize repeated fragmentation of abundant precursor ions.

Instrument mass calibration was performed before sample acquisition, and a solvent blank was included in the analytical sequence. No dedicated pooled quality-control sample was prepared or injected.

### 2.7 Feature-based molecular networking and metabolite annotation

#### 2.7.1 MS data processing

Raw UHPLC–Orbitrap–HRMS/MS data acquired using polarity switching were converted to centroided mzML format using MSConvert within ProteoWizard, with vendor peak picking and positive-polarity filtering enabled. Only positive-ion scans were retained for subsequent MS-DIAL processing and FBMN analysis.

The *Diaporthe* sp. PBS30-7 mzML file was processed together with that of PBS24-3, a separate *Diaporthe* isolate included solely as the alignment reference, using MS-DIAL v4.9.221218. Feature detection, MS/MS spectral deconvolution, and isotope and adduct annotation were performed using MS1 and MS2 mass tolerances of 0.01 and 0.025 Da, respectively. Feature detection was conducted using a minimum peak height of 1,000 amplitude units and a mass-slice width of 0.1 Da. Chromatographic signals were smoothed using a linear weighted moving-average algorithm over three scans, and the minimum peak width was set to five scans. No exclusion-mass list was applied.

MS/MS spectral deconvolution was performed using a sigma-window value of 0.5 and an MS/MS abundance cutoff of 0 amplitude units. Fragment ions above the precursor *m/z* were excluded. Isotopic ions were retained within 0.5 Da, whereas the option to retain isotopic ions without MS2Dec processing was disabled. The positive-ion adducts considered were [M+H]^+^, [M+NH4]^+^, [M+Na]^+^, and [M+K]^+^.

Spectral-library matching was performed against the MSMS-Public positive-ion spectral library (VS19), using mass tolerances of 0.01 Da for precursor ions and 0.05 Da for fragment ions. A library-matching score cutoff of 80% and a relative-abundance cutoff of 0% were applied. Retention time was not used for library scoring or filtering, and no external text-based spectral library was used. Because authentic standards were not analyzed, library matches were treated as putative annotations.

Peak alignment was performed using the PBS24-3 dataset as the reference, with retention-time and MS1 tolerances of 0.05 min and 0.015 Da, respectively. The retention-time and MS1 similarity factors were each set to 0.5. The peak-count filter and minimum within-group detection threshold were both set to 0%. Forced gap filling was enabled, whereas blank-based feature removal was not applied.

Following peak alignment, only the *Diaporthe* sp. PBS30-7 data were exported from MS-DIAL using its integrated GNPS export function. This generated a coordinated MGF spectral file and TXT feature quantification table, preserving correspondence between the feature identifiers in the TXT file and the SCANS identifiers in the MGF file. These files, together with the corresponding *Diaporthe* sp. PBS30-7 mzML file, were uploaded to GNPS.

#### 2.7.2 Feature-based molecular networking

Feature-based molecular networking was performed on the positive-ion data using the standard FBMN workflow on the Global Natural Products Social Molecular Networking platform (Nothias et al., 2020). The complete GNPS job and associated parameters are publicly accessible under Task ID: 1195aa31edf84e3eb6b7da678c56d321.

Before network construction, fragment ions within ±17 Da of the precursor m/z were removed. The MS/MS spectra were then window-filtered by retaining the six most intense fragment ions within each ±50-Da window. The precursor- and fragment-ion mass tolerances were both set to 0.02 Da.

Network edges were retained when the paired nodes shared a cosine similarity score of at least 0.7 and a minimum of six matched fragment ions. Edges were further required to satisfy the reciprocal top-10 criterion, whereby each node occurred among the 10 most similar nodes of its paired node. Molecular-family size was limited to 100 nodes through iterative removal of the lowest-scoring edges.

Spectral-library matching was performed against the GNPS reference libraries using the same precursor- and fragment-ion filtering criteria. Only library matches with cosine similarity scores of at least 0.7 and a minimum of six matched fragment ions were retained. Additional putative annotations were generated using DEREPLICATOR. The resulting molecular network was visualized and manually inspected using Cytoscape v3.10.4.

### 2.8 Molecular docking

To investigate potential molecular targets associated with the metabolite families identified by FBMN, representative compounds were selected from the annotated beauvericin and diketopiperazine (DKP) families based on structural similarity and previous reports of related fungal metabolites for molecular docking against representative fungal proteins involved in cell wall biosynthesis, membrane homeostasis, stress adaptation, and multidrug resistance.

Potential protein targets were selected based on their established roles in fungal physiology and antifungal therapy (Table 1). Protein sequences were retrieved from UniProt and experimentally determined 3D structures were obtained from the Protein Data Bank (PDB) as references. All database records were accessed on 27 March 2026. All protein structures were generated using AlphaFold or homology modeling and therefore lacked co-crystallized ligands for cognate redocking validation. Protonation states were assigned at pH 7.0 during system preparation using CHARMM-GUI. And the prepared receptor structures were converted to PDBQT format using the “vina Wizard” module in PyRx. Binding sites were defined using the coordinates of co-crystallized ligands or co-factors when available or inferred from homologous protein-ligand complexes and experimentally characterized functional residues for proteins lacking resolved structures, as shown in Table 1.

**Table 1:** Representative fungal protein targets selected for molecular docking based on their established biological functions.

Target	Biological function	Relevance to antifungal therapy	UniProt ID	Binding site reference
Sterol 14α-demethylase (CYP51/Erg11)	Catalyzes a key step in ergosterol biosynthesis and is essential for fungal membrane integrity	One of the best-validated antifungal targets and the primary target of azole drugs	A0A9W9NJZ0	5TL8
β-1,3-Glucan synthase (Fks1)	Responsible for the synthesis of β-1,3-glucan, a major structural component of the fungal cell wall	Canonical target of echinocandin antifungals and essential for cell wall integrity	A0A9W9TIU5	9PE2
Squalene epoxidase (Erg1)	Catalyzes the conversion of squalene to 2,3-oxidosqualene in the ergosterol biosynthetic pathway	Molecular target of allylamine antifungals and an upstream regulator of sterol biosynthesis	A0A9W9NL69	6C6P (chain A)
Heat shock protein 90 (Hsp90)	Molecular chaperone involved in protein folding, stability, and stress response	Plays an important role in antifungal tolerance, resistance, and stress adaptation	A0A9W9NQ59 (residues 10–211)	6CJS (chain A)
ABC transporter (ABC)	ATP-dependent membrane transporter involved in substrate efflux and multidrug resistance	Closely associated with fungal drug resistance and reduced intracellular accumulation of antifungal compounds	A0A9W9NYB0; homology model built in SWISS-MODEL	3G60 (chain A)

Ligand structures were generated via parsing SMILES strings using RDKit (Rappe et al., 1992), followed by the addition of explicit hydrogen atoms, 3D conformer generation using ETKDGv3 with a fixed random seed of 1, and geometry optimization using the UFF force field. The optimized hydrogen-containing structures were exported in PDB format and converted to AutoDock Vina-compatible PDBQT files using Open Babel (O'Boyle et al., 2011). During PDBQT preparation, non-polar hydrogens were merged with their attached carbon atoms, whereas polar hydrogens were retained explicitly.

Molecular docking was carried out with AutoDock Vina v1.1.2 (Trott and Olson, 2010) using an exhaustiveness value of 64 and a fixed random seed of 42 to ensure reproducibility. For each ligand–protein pair, the highest-ranked binding pose was retained for subsequent analysis of predicted binding affinity and protein–ligand interactions. Molecular docking was conducted as an exploratory analysis to infer broad binding trends and prioritize potential target families, rather than for quantitative structural refinement.

### 2.9 Statistical analysis

Quantitative experimental data are presented as the mean ± standard deviation (SD) of three independent biological replicates. Statistical analyses were performed using GraphPad Prism v10.5.0 (GraphPad Software, San Diego, CA, USA). Comparisons between two groups were performed using an unpaired two-tailed Student's *t*-test, whereas multiple-group comparisons were analyzed by one-way analysis of variance (ANOVA) followed by Tukey's multiple comparison test, where appropriate. Differences were considered statistically significant at *P* < 0.05. Molecular docking results were analyzed descriptively and were not subjected to statistical hypothesis testing.

## 3 Results

### 3.1 *Diaporthe* sp. PBS30-7 exhibits condition-dependent growth across temperature, nutrient, and pH gradients

Temperature and culture medium influenced the growth of *Diaporthe* sp. PBS30-7 in distinct ways, as assessed by standardized colony expansion, growth kinetics estimated using the modified Gompertz model, and colony morphology under different environmental conditions (Figure 1). Two-way ANOVA showed that culture medium explained the largest proportion of the variation in colony growth (58.34%), followed by temperature (19.89%) and the temperature × medium interaction (18.50%).

**Figure 1:**
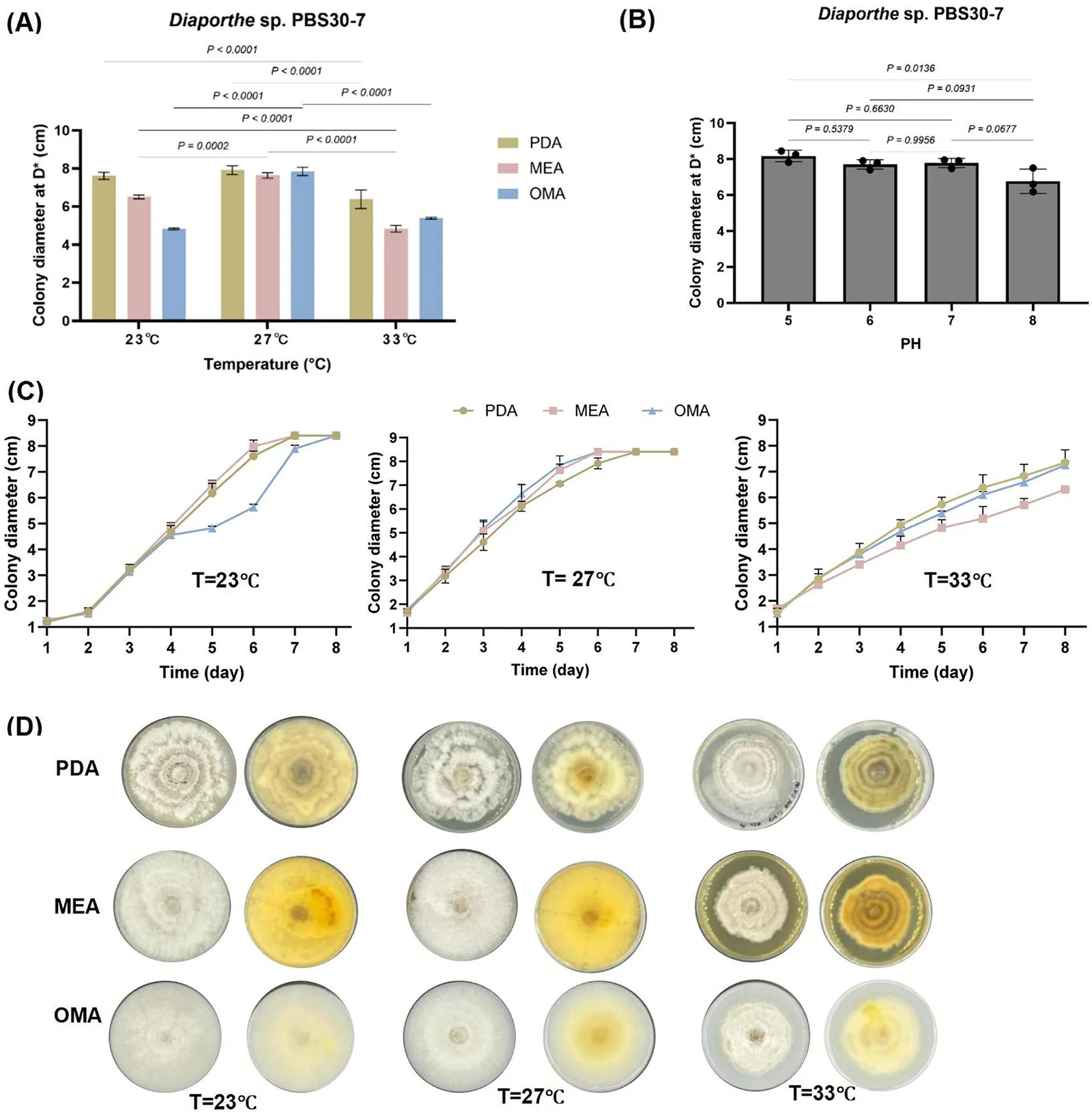
Physiological responses of *Diaporthe* sp. PBS30-7 under environmental gradients. **(A)** Colony diameter of *Diaporthe* sp. PBS30-7 after 6 days of incubation on PDA, MEA, and OMA under three temperature conditions (23 °C, 27 °C, and 33 °C). **(B)** Colony diameter of *Diaporthe* sp. PBS30-7 after 7 days of incubation under different pH conditions. **(C)** Radial growth kinetics of *Diaporthe* sp. PBS30-7 under the same temperature–medium combinations, showing colony expansion over time. **(D)** Representative colony morphology of *Diaporthe* sp. PBS30-7 after 6 days of incubation on PDA, MEA, and OMA under different temperature conditions.

Colony expansion was generally greater at 23 and 27 °C than at 33 °C (Figure 1A). On PDA, colony diameters at 33 °C were significantly smaller than those at both 23 and 27 °C (both *P* < 0.00001), whereas no significant difference was detected between 23 and 27 °C (*P* = 0.3877). A similar pattern was observed on MEA, where colony expansion declined significantly at 33 °C compared with both lower temperatures (both *P* < 0.00001), although colony diameter also differed significantly between 23 and 27 °C (*P* = 0.0002). On OMA, colonies reached their largest diameter at 27 °C and were significantly larger than those at both 23 and 33 °C (both *P* < 0.00001), whereas colony diameters at 23 and 33 °C did not differ significantly at the standardized endpoint (*P* = 0.052). Extended observations beyond the standardized endpoint showed that colonies grown on OMA at 23 °C completely covered the 90-mm plates after an additional 2 days, whereas colonies grown at 33 °C reached a mean diameter of 7.19 cm. These observations are provided for descriptive comparison only and were not included in the statistical analysis.

Time-course analysis revealed distinct growth trajectories among temperature–medium combinations (Figure 1C). The modified Gompertz model provided an excellent fit to the observed growth curves (*R*^2^ = 0.975–0.999), and the corresponding kinetic parameters are summarized in Table 2. At 27 °C, colonies entered the exponential growth phase after a short lag period and exhibited similar expansion trajectories on PDA, MEA, and OMA, with comparable lag-phase durations and consistently high maximum growth rates. At 23 °C, growth trajectories diverged among media despite similar growth initiation, with colonies on OMA exhibiting a lower maximum growth rate than those on PDA and MEA.

**Table 2:** Growth kinetic parameters of *Diaporthe* sp. PBS30-7 under different temperature and culture medium conditions estimated using the modified Gompertz model.

Temperature	Medium	*μ_*max*_*(cm/day)	λ(day)	*R* ^2^
23 °C	PDA	1.625 ± 0.05*^*a,x*^*	1.418 ± 0.14*^*a,x*^*	0.9927
	MEA	1.709 ± 0.04*^*a,x*^*	1.467 ± 0.01*^*a,x*^*	0.9987
	OMA	1.270 ± 0.05*^*b,y*^*	0.9329 ± 0.05*^*b,x*^*	0.9963
27 °C	PDA	1.586 ± 0.10*^*a,x*^*	0.349 ± 0.08*^*a,y*^*	0.9958
	MEA	1.751 ± 0.40*^*a,x*^*	0.383 ± 0.29*^*a,y*^*	0.9928
	OMA	1.846 ± 0.19*^*a,x*^*	0.443 ± 0.06*^*a,y*^*	0.9945
33 °C	PDA	1.197 ± 0.03*^*a,y*^*	0.134 ± 0.21*^*a,y*^*	0.9752
	MEA	0.893 ± 0.10*^*a,z*^*	0.008 ± 0.01*^*b,z*^*	0.9987
	OMA	1.118 ± 0.03*^*a,y*^*	0.023 ± 0.14*^*a,z*^*	0.9924

Data are presented as mean ± SD (*n* = 3). Different superscript letters indicate significant differences as determined by Tukey's HSD test (*p* < 0.05). Lowercase letters (a, b): compare different media within the same temperature (i.e., within a row). Values sharing the same letter are not significantly different. Uppercase letters (x, y, z): compare different temperatures within the same medium (i.e., within a column). Values sharing the same letter are not significantly different. μ_max_ = maximum specific growth rate (cm/day), λ = lag phase duration (day), *R*^2^ = coefficient of determination, showing the goodness of fit of the Gompertz model; values closer to 1 represent more reliable fits.

Colony morphology varied with both temperature and nutrient composition (Figure 1D). At 27 °C, colonies were compact and produced abundant aerial mycelia, particularly on PDA. Colonies on MEA showed a similarly compact morphology, whereas those on OMA exhibited more diffuse colony margins. At 23 °C, colonies on all three media displayed a looser mycelial organization. Radial expansion was reduced at 33 °C, and colony pigmentation varied among media.

Compared with temperature and culture medium, environmental pH had a smaller influence on colony expansion (Figure 1B). Colony growth was similar at pH 5 and pH 7, whereas a modest but significant reduction was observed at pH 8 (*P* < 0.05).

### 3.2 Competitive interactions between *Diaporthe* sp. PBS30-7 and two *Penicillium* species

Dual-culture assays demonstrated that *Diaporthe* sp. PBS30-7 consistently maintained colony expansion while restricting the growth of both *Penicillium sumatrense* and *P. citrinum*, forming a Type F interaction pattern with each pathogen (Figure 2A). Against *P. sumatrense*, colony diameter was reduced from 3.07 cm in monoculture to 1.33 cm in dual culture by Day 8, corresponding to a mean mycelial growth inhibition (MGI) of 65.14 ± 2.29% (Figures 2B, C). During the first 3 days of incubation, both fungi exhibited progressive radial expansion. Thereafter, expansion of *P. sumatrense* became minimal, whereas *Diaporthe* sp. PBS30-7 continued to expand from 1.32 cm on Day 3 to 5.30 cm by Day 8.

**Figure 2:**
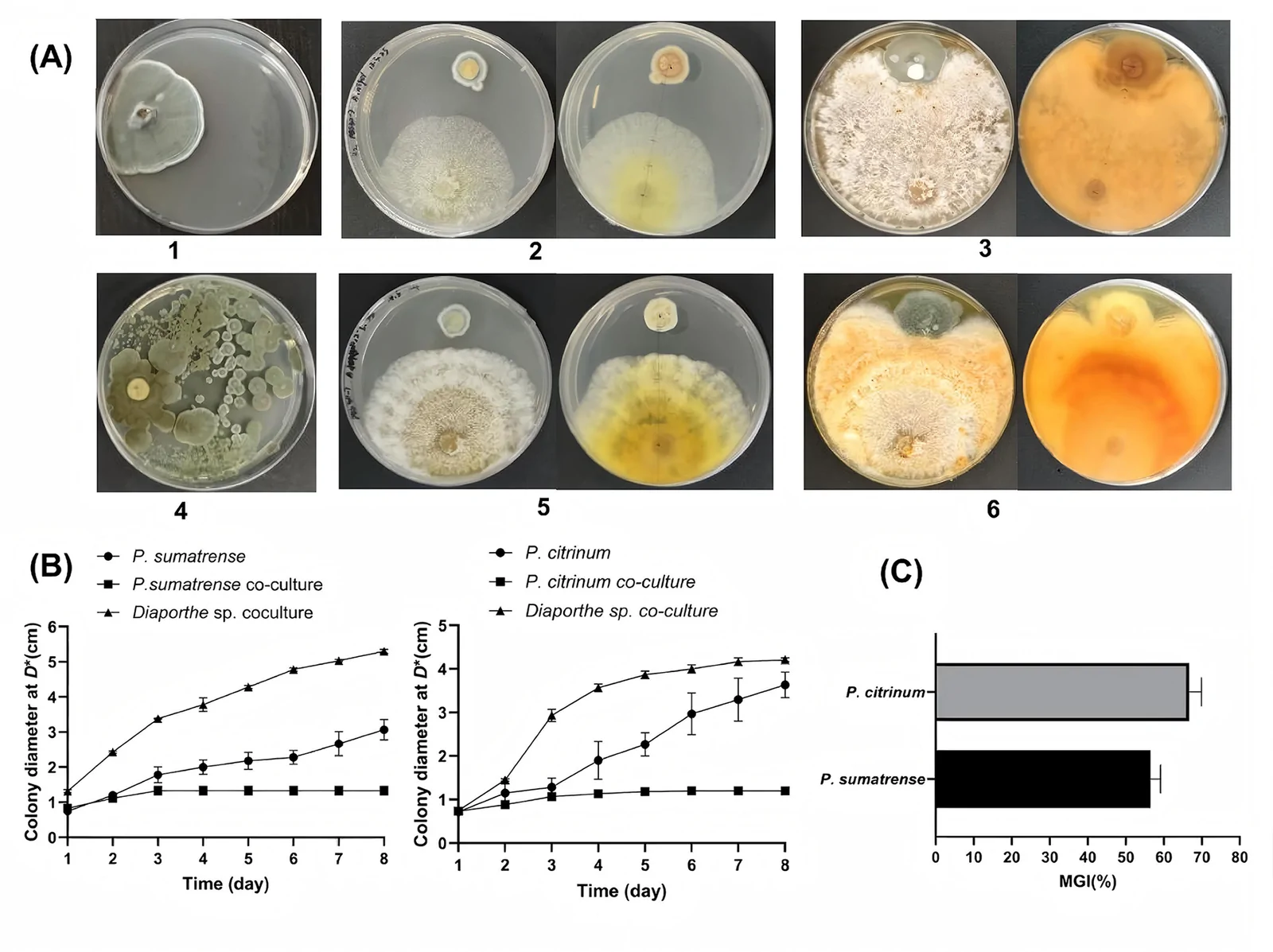
Direct confrontation between *Diaporthe* sp. PBS30-7 and two *Penicillium* pathogens. **(A)** Representative monoculture controls and dual-culture plates. Panels 1–3 show *P. sumatrense*, and panels 4–6 show *P. citrinum*. Panels 1 and 4 show the corresponding *Penicillium* monoculture controls after 8 days; panels 2 and 5 show dual cultures after 3 days; and panels 3 and 6 show dual cultures after 8 days, when Type F interactions were evident. **(B)** Temporal changes in colony diameter of *Diaporthe* sp. PBS30-7 and the two *Penicillium* species during dual culture. **(C)** Mycelial growth inhibition (MGI, %) of *P. sumatrense* and *P. citrinum* calculated from colony diameters at Day 8. Bars represent mean ± SD (*n* = 3).

A comparable competitive pattern was observed against *P. citrinum*. Colony diameter decreased from 3.63 cm in monoculture to 1.20 cm in dual culture by Day 8, corresponding to an MGI of 56.70 ± 1.42% (Figures 2B, C). Radial expansion of *P. citrinum* became minimal after approximately Day 5, whereas *Diaporthe* sp. PBS30-7 continued expanding throughout the incubation period and reached a colony diameter of 4.19 cm by Day 8.

Microscopic examination further distinguished the interaction zones from the corresponding control cultures (Figure 3). In the absence of *Diaporthe* sp. PBS30-7, both *P. sumatrense* and *P. citrinum* produced intact hyphae and normally developed conidiophores. In contrast, dual-culture interaction with *Diaporthe* sp. PBS30-7 was associated with distinct morphological alterations in both pathogens. In *P. sumatrense*, conidiophores were deformed, localized pigment accumulation was evident, and *Diaporthe* sp. PBS30-7 hyphae overgrew and formed extensive hyphal interweaving within the interaction zone. In *P. citrinum*, hyphae were deformed and accompanied by reduced conidiation together with localized hyphal lysis.

**Figure 3:**
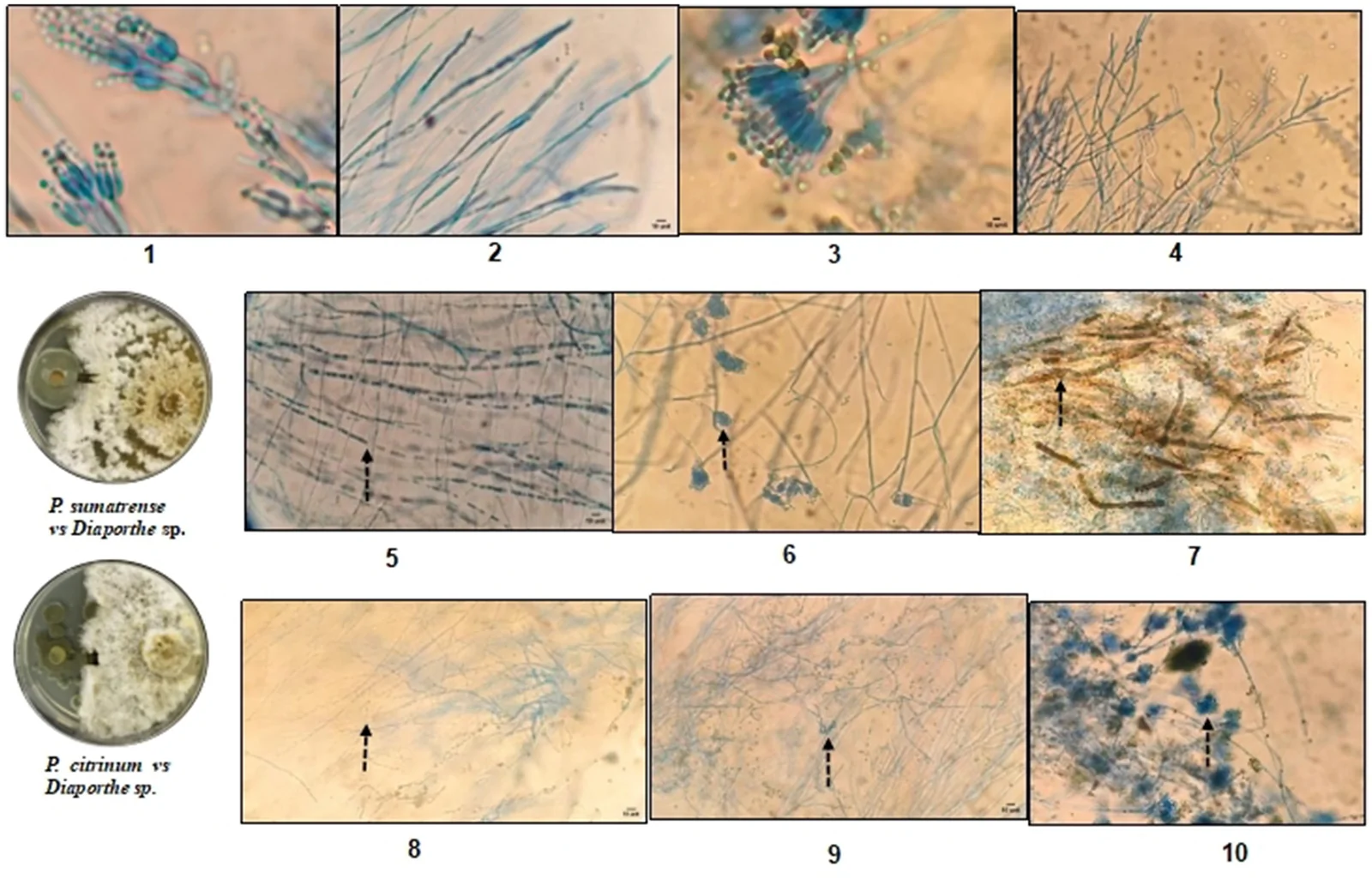
*Diaporthe* sp. PBS30-7 and two *Penicillium* pathogens during dual culture. Panels 1–4 show untreated controls of *P. sumatrense* (1–2) and *P. citrinum* (3–4), displaying intact hyphae and normal conidiophore development. Panels 5–7 show *P. sumatrense* in dual culture with PBS30-7, showing hyphal contact at the interaction interface, partial hyphal lysis, abnormal conidiophore morphology, and localized pigment accumulation (arrows). Panels 8–10 show *P. citrinum* in dual culture with *Diaporthe* sp. PBS30-7, showing hyphal deformation, partial hyphal lysis, and reduced conidiation (arrows).All micrographs were obtained after 8 days of incubation at 27 °C using light microscopy (400×). Scale bars = 10 μm.

### 3.3 Cell-free culture filtrates of *Diaporthe* sp. PBS30-7 retain antifungal activity

Cell-free culture filtrates prepared from 7-day liquid cultures of *Diaporthe* sp. PBS30-7 retained measurable antifungal activity against both *Penicillium sumatrense* and *P. citrinum* in the absence of living mycelia and direct fungal contact (Figure 4A). The culture filtrate inhibited mycelial growth of both pathogens, with mean MGI values of 41.32% for *P. sumatrense* and 45.12% for *P. citrinum* (mean ± SD, *n* = 4). Two-way ANOVA followed by Tukey's multiple-comparison test detected significant differences among treatments. Under the same experimental conditions, itraconazole produced higher inhibition, with MGI values of 54.91% and 77.41%, respectively.

**Figure 4:**
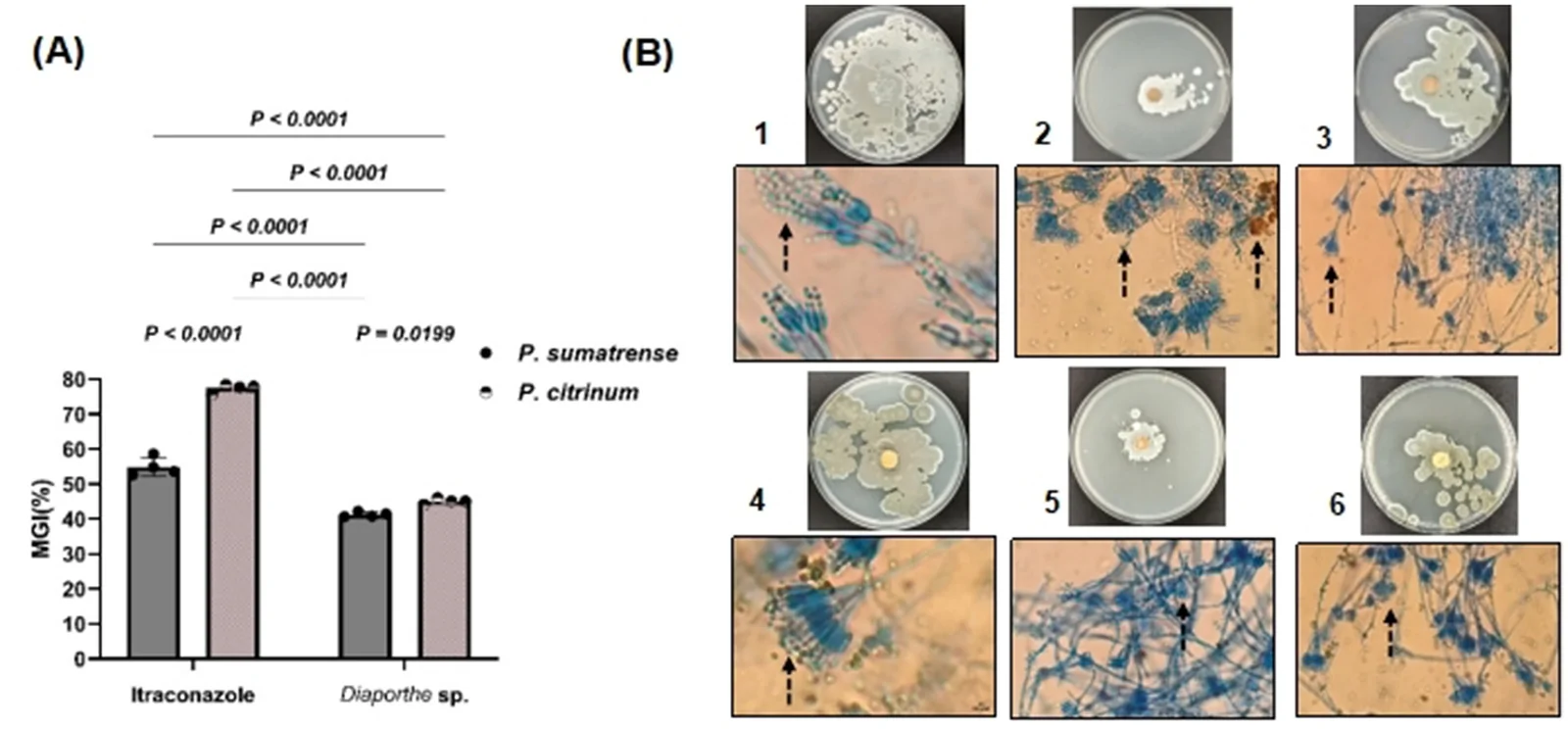
Antifungal activity of *Diaporthe* sp.PBS30-7 culture filtrate against *Penicillium* pathogens. **(A)** Mycelial growth inhibition (MGI, %) of *Penicillium sumatrense* and *P. citrinum* after exposure to a 7-day culture filtrate of *Diaporthe* sp.PBS30-7. Itraconazole was included as a positive antifungal control. Bars represent mean ± SD (*n* = 4). Statistical significance was determined using two-way ANOVA followed by Tukey's multiple comparisons test. **(B)** Colony morphology (upper panels) and corresponding microscopic observations (lower panels) of *P. sumatrense* (panels 1–3) and *P. citrinum* (panels 4–6) under three experimental conditions: untreated control (panels 1, 4), itraconazole-treated positive control (panels 2, 5), and exposure to *Diaporthe* sp. culture filtrate (panels 3, 6).

Morphological responses differed among treatments (Figure 4B). Compared with the untreated controls, *P. sumatrense* exposed to the culture filtrate exhibited altered conidiophore morphology together with reduced conidiation, whereas *P. citrinum* primarily showed reduced conidiation. Treatment with itraconazole resulted in greater inhibition of colony growth and more pronounced morphological alterations than those observed following exposure to the culture filtrate.

### 3.4 Metabolomic profiling reveals chemically diverse candidate metabolite families in *Diaporthe* sp. PBS30-7

Untargeted UHPLC–Orbitrap HRMS/MS coupled with feature-based molecular networking (FBMN) resolved the metabolite profile of *Diaporthe* sp. PBS30-7 into multiple structurally related molecular families (Supplementary Figure 1). Spectral similarity organized molecular features into discrete clusters, among which beauvericin-type cyclodepsipeptides and diketopiperazine (DKP)-related metabolites represented two of the most prominent molecular families. These clusters exhibited coherent network connectivity together with characteristic MS/MS fragmentation patterns and were therefore examined in greater detail. A representative base-peak chromatogram is provided in Supplementary Figure 2.

The beauvericin molecular family comprised a series of closely connected features sharing conserved product ions at m/z 134.0963 and 91.0546 (Figure 5A). The cluster contained beauvericin together with several structurally related analogs, including beauvericin A, B, C, F, and a beauvericin K-type feature (Figure 5B). Beauvericin was detected as the sodium adduct ([M+Na]^+^, m/z 806.3984), whereas beauvericin A was represented by both protonated ([M+H]^+^, m/z 798.4312) and ammonium ([M+NH4]^+^, m/z 815.4597) ions. Additional analogs occupied adjacent positions within the same molecular family and displayed highly similar fragmentation behavior. One feature assigned the same molecular formula as beauvericin A exhibited a distinct MS/MS fragmentation profile and was therefore tentatively annotated as beauvericin F. A lower-mass feature within the same cluster was tentatively assigned as a beauvericin K-type compound based on accurate mass together with its spectral similarity to the remaining members of the family.

**Figure 5:**
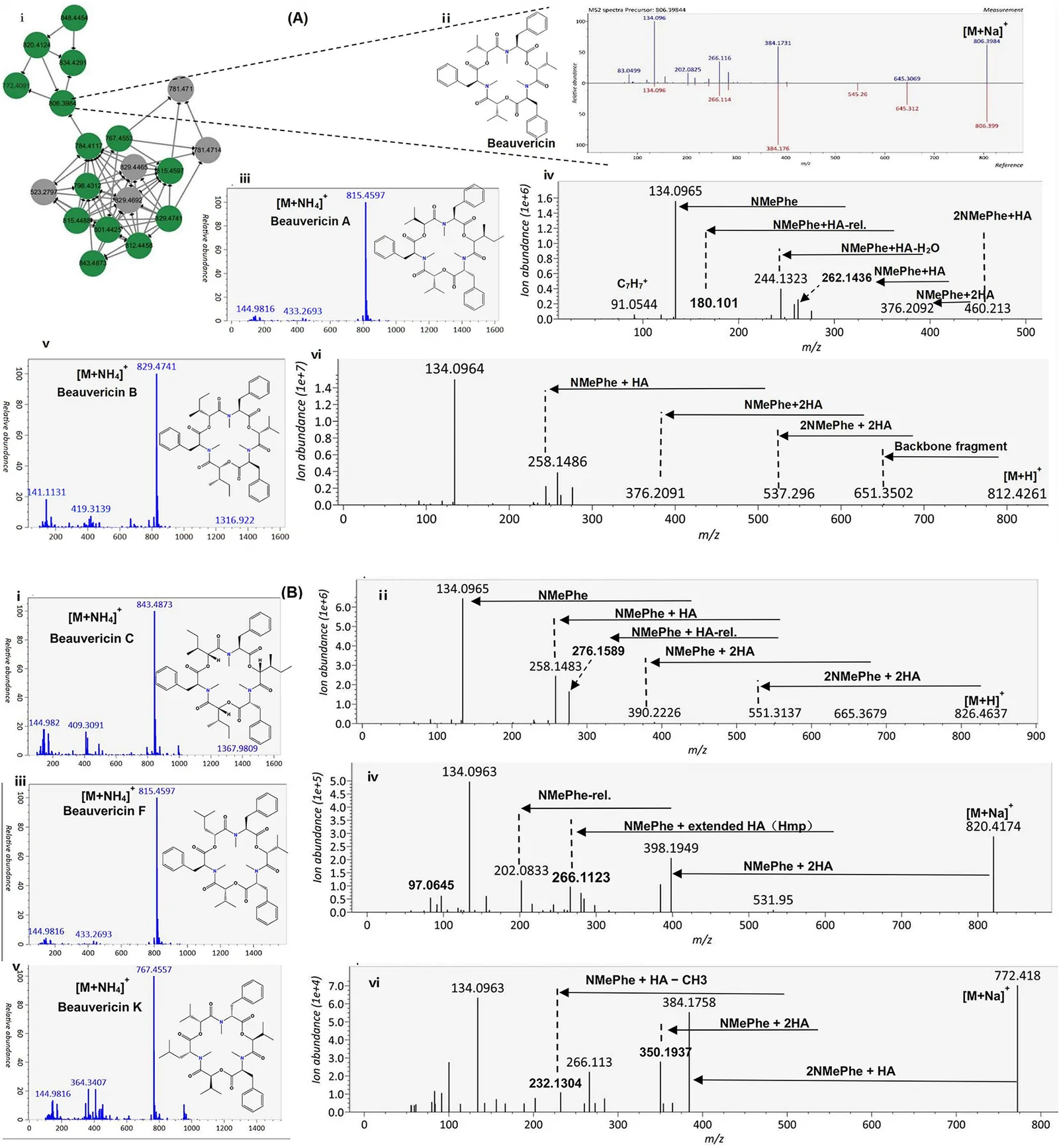
Molecular networking and MS/MS-based annotation of beauvericin-type metabolites from *Diaporthe* sp. PBS30-7. **(A)** Beauvericin-related molecular network and spectral evidence supporting the putative annotation of beauvericin, beauvericin A, and beauvericin B. **(B)** Precursor-ion and annotated MS/MS fragmentation spectra supporting the putative annotation of beauvericins C, F, and K.

A second prominent molecular family comprised DKP-related metabolites (Figure 6). Spectra within this cluster showed recurrent fragmentation characteristics, including a product ion at m/z 70.0656 and, in selected features, an additional ion at m/z 136.0754. Neutral losses consistent with dehydration were also observed in several hydroxylated members of the cluster. Based on accurate mass and MS/MS fragmentation, tentative annotations within the DKP-related family included cyclo(D-Val-L-Pro), cyclo(Leu-Pro), cyclo(Pro-Thr), cyclo(L-Pro-4-hydroxy-L-Leu), and cyclo(Tyr-Pro). Additional related features were tentatively assigned as prolylphenylalanine and 3-(1H-indol-3-ylmethyl)-2,3,6,7,8,8a-hexahydropyrrolo[1,2-a]pyrazine-1,4-dione. Collectively, these results demonstrate that the extracellular metabolome of *Diaporthe* sp. PBS30-7 comprises multiple structurally related candidate metabolite families spanning cyclodepsipeptides and diketopiperazines, providing a chemically diverse framework for subsequent analyses.

**Figure 6:**
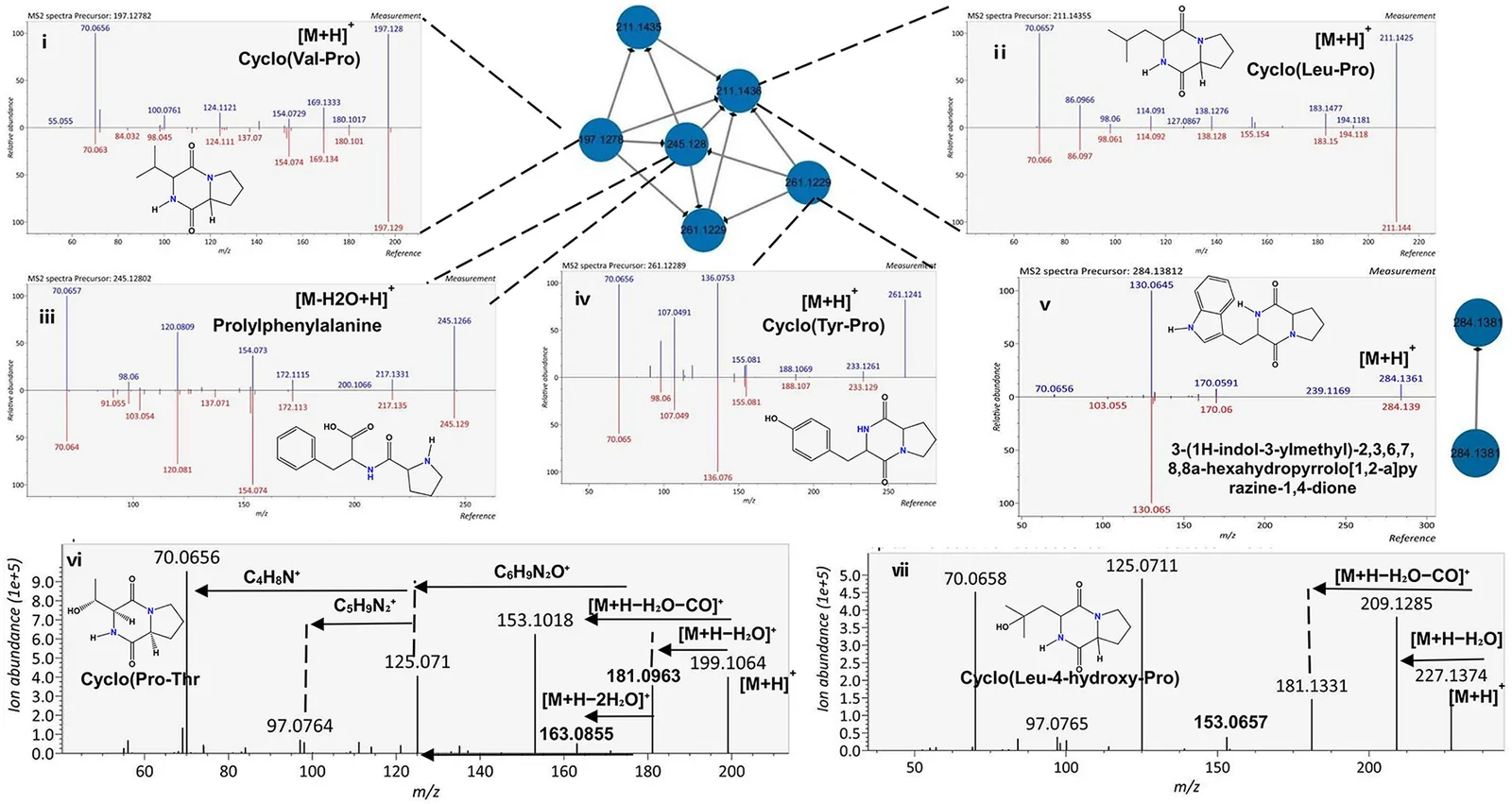
Molecular networking and MS/MS-based annotation of diketopiperazine-type metabolites from *Diaporthe* sp. PBS30-7. The molecular network and spectral-library mirror plots support the putative annotation.

### 3.5 Docking-based prioritization of potential fungal protein targets

Molecular docking revealed distinct target-dependent binding profiles across the annotated metabolites (Figure 7), with the complete docking results provided through a supplementary data link in the Data Availability. Overall, beauvericin derivatives exhibited the most prominent docking profiles, with multiple analogs showing favorable docking scores against CYP51, Fks1, and the ABC transporter, highlighting these proteins as candidate targets warranting further investigation. At the individual-compound level, Beauvericin K showed the most favorable docking score against CYP51 (−11.1 kcal/mol), Beauvericin H2 showed a favorable score against Fks1 (−10.5 kcal/mol), and Beauvericin C showed a favorable score against the ABC transporter (−9.1 kcal/mol). In comparison, the DKPs generally exhibited more moderate docking profiles. Among them, cyclo(L-Tyr-L-Tyr) was the most notable candidate, showing relatively favorable scores across multiple targets, with a score of −8.6 kcal/mol against Hsp90. Erg1 generally yielded only weak-to-moderate docking scores (−4.7 to −6.4 kcal/mol) among compounds for which valid docking poses were obtained and were therefore not prioritized for subsequent structural analysis. Taken together, these family- and compound-level docking patterns highlighted beauvericin derivatives as the principal candidate metabolite family for further investigation. Accordingly, Beauvericin K–CYP51, Beauvericin H2–Fks1, Beauvericin C–ABC transporter, and cyclo(L-Tyr-L-Tyr)–Hsp90 were selected as representative high-ranking ligand–target pairs for binding-pose analysis (Figure 8).

**Figure 7:**
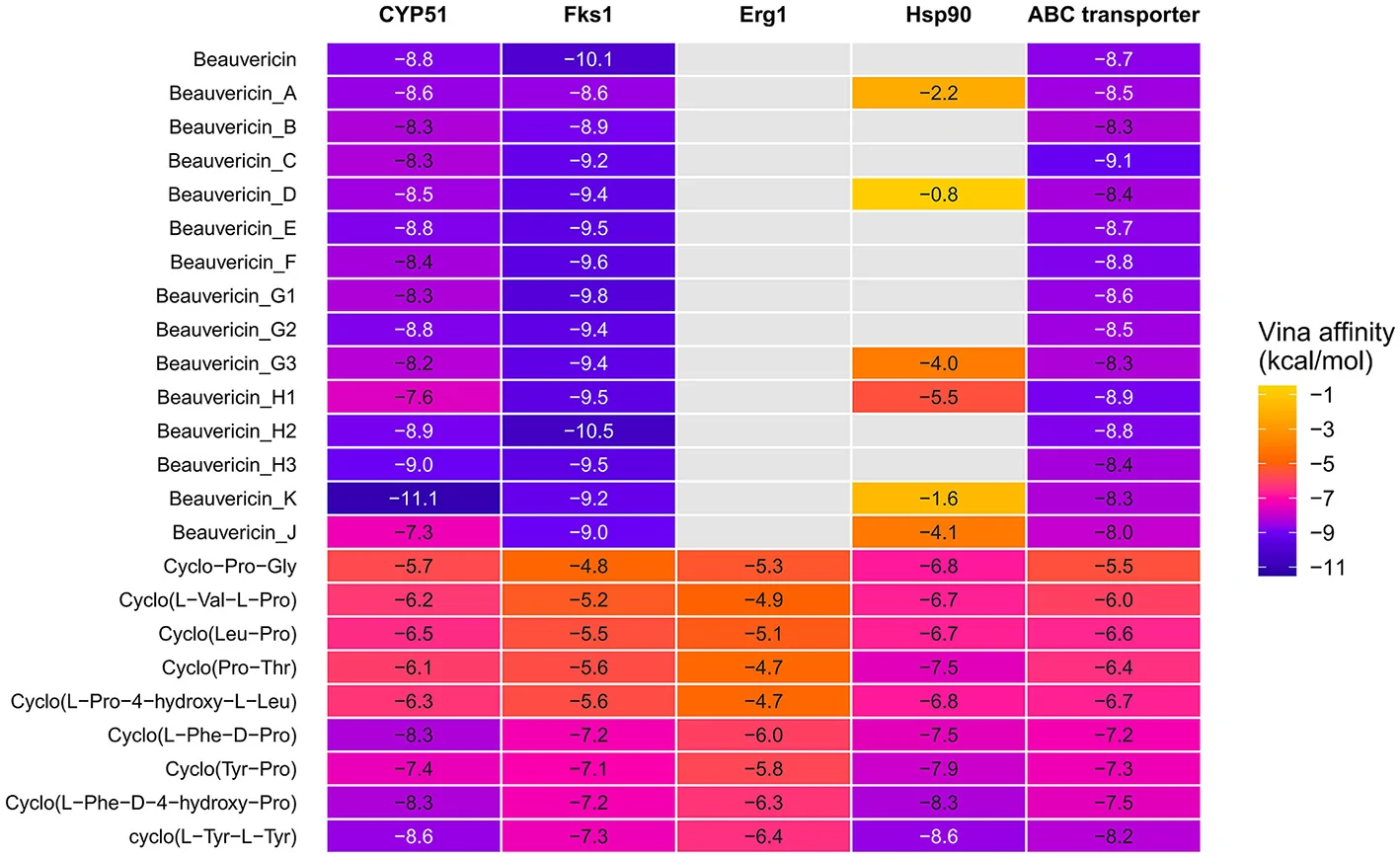
Heat map of predicted molecular docking affinities of selected beauvericin derivatives and cyclic dipeptides against five fungal targets: CYP51, Fks1, Erg1, Hsp90, and an ABC transporter. Values shown in each cell are AutoDock Vina binding affinities (kcal/mol), with more negative values indicating stronger predicted binding. The color ranges from yellow (weaker predicted binding) to dark purple (stronger predicted binding). Gray cells indicate that no valid docking score was obtained for that ligand-target pair.

**Figure 8:**
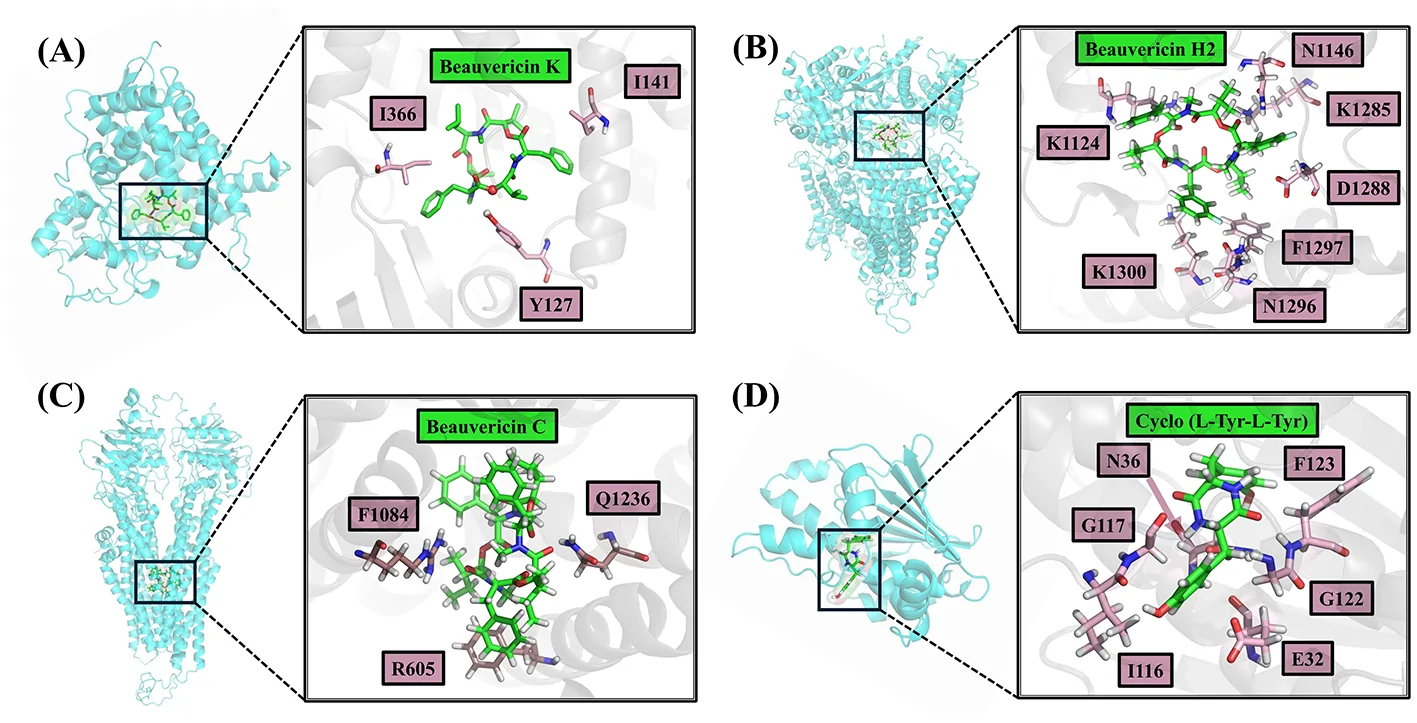
Docking-derived binding modes of selected fungal targets with candidate antifungal metabolites. **(A)** Beauvericin K in the CYP51 binding pocket, featuring a Y127-O5 hydrogen bond and pi-sigma contacts involving I141 and I366. **(B)** Beauvericin H2 in Fks1, showing a halogen contact with N1296, hydrogen bonds with K1124, N1146, and K1285, and aromatic contacts involving D1288, K1300, and F1297. **(C)** Beauvericin C in the ABC transporter, forming hydrogen bonds with Q1236 and R605 and a pi-pi stacking contact with F1084. **(D)** cyclo(L-Tyr-L-Tyr) in Hsp90, anchored by hydrogen bonds involving N36, I116, G122, and F123; its phenyl ring is clamped between E32 and G117. Protein cartoons, ligands, and key residues are shown in cyan, green, and pink, respectively.

Representative docking poses further illustrated the predicted binding environments of the four prioritized ligand–target pairs (Figure 8). Beauvericin K occupied the predicted CYP51 binding pocket, where its docking pose involved a hydrogen bond with Y127 and additional contacts with I141 and I366. Beauvericin H2 was accommodated within the predicted Fks1 binding region and interacted with multiple surrounding residues, including K1124, N1146, K1285, D1288, N1296, F1297, and K1300. Within the ABC transporter, Beauvericin C was positioned in proximity to R605, F1084, and Q1236, with its predicted pose involving hydrogen-bonding and aromatic interactions. Cyclo(L-Tyr-L-Tyr) occupied a localized binding region of Hsp90 surrounded by residues including E32, N36, I116, G117, G122, and F123, with the predicted pose displaying multiple polar and aromatic interactions.

## 4 Discussion

This study integrates physiological characterization, time-resolved fungal confrontation assays, cell-free culture-filtrate testing, and exploratory chemical profiling to characterize *Diaporthe* sp. PBS30-7. Previous studies have separately examined the environmental responses, antagonistic activity, culture-filtrate bioactivity, and secondary-metabolite production of *Diaporthe* spp.; here, these dimensions were evaluated within a single endophytic isolate while maintaining a clear distinction between phenotypic association and mechanistic causation.

### 4.1 Environmental responsiveness provides physiological context for competitive interactions

The growth profile of *Diaporthe* sp. PBS30-7 indicates a predominantly mesophilic but nutrient-dependent physiological response. Its thermal pattern is broadly consistent with that reported across *Diaporthe*, for which optimal mycelial growth generally occurs between approximately 24 and 30 °C (Ariyawansa et al., 2021; Francia et al., 2025). By contrast, the reduced growth of *Diaporthe* sp. PBS30-7 under mildly alkaline conditions, compared with its relatively stable performance under moderately acidic to neutral conditions, points to strain-level variation in pH tolerance. Comparable variability has been reported among tea-associated *Diaporthe* isolates, indicating that environmental responses cannot be inferred reliably from genus-level characteristics alone (Ariyawansa et al., 2021).

The significant temperature–medium interaction further shows that the thermal performance of *Diaporthe* sp. PBS30-7 depended on nutritional context. Differences among media were attenuated at 27 °C but became more pronounced at 23 and 33 °C, with PDA supporting greater colony expansion under the latter conditions. This conditional response is consistent with the species- and medium-dependent growth reported for other *Diaporthe* taxa cultivated on PDA, MEA, and OMA (Tsoukas et al., 2025). However, the present experiments did not identify the nutritional components or physiological processes responsible for these differences. Moreover, because growth was evaluated in monoculture, environmental responsiveness and rapid colony expansion cannot be equated with antagonistic capacity or competitive fitness. These findings instead define the cultivation conditions that support vegetative establishment by *Diaporthe* sp. PBS30-7 and provide the physiological context for interpreting its subsequent interactions with the two *Penicillium* species.

### 4.2 Competitive interactions reflect multiple complementary processes

Time-resolved confrontation assays showed that the competitive phenotype of *Diaporthe* sp. PBS30-7 developed progressively and varied between the two pathogens. Growth of *P. sumatrense* was restricted earlier and ultimately to a greater extent than that of *P. citrinum*, indicating that endpoint measurements alone do not fully capture the dynamics of fungal confrontation. Previous studies of antagonistic endophytic *Diaporthe* strains have largely relied on endpoint dual-culture assessments (Verma et al., 2022). The contrasting trajectories observed here may reflect differences in the growth dynamics or susceptibility of the two pathogens, although their physiological basis remains unresolved.

Morphological changes at the interaction interface further suggest that several ecological processes may operate concurrently. During confrontation with *P. sumatrense, Diaporthe* sp. PBS30-7 overgrew the pathogen and formed extensive hyphal intermingling, whereas *P. citrinum* exhibited reduced conidiation, hyphal deformation, and localized lysis. Hyphal overgrowth and intermingling can occur during interference competition but may also accompany mycoparasitic interactions. Mycoparasitism by *D. eres* was recently demonstrated through more definitive microscopic evidence, including host-hypha colonization, attachment structures, and putative penetration structures (Geiger et al., 2026). *Diaporthe* sp. PBS30-7 showed no comparable evidence of penetration or intracellular colonization; therefore, its overgrowth cannot be interpreted as definitive mycoparasitism. Likewise, the deformation and lysis of *P. citrinum* hyphae are consistent with cellular injury but could arise from diffusible inhibitory constituents, contact-associated damage, or localized nutrient limitation.

Cell-free culture filtrates remained inhibitory to both *Penicillium* species after removal of living *Diaporthe* sp. PBS30-7 mycelia, providing complementary evidence for extracellular constituents capable of contact-independent antifungal activity. The filtrate also altered conidiophore morphology and reduced conidiation in *P. sumatrense*, whereas reduced conidiation was the predominant response of *P. citrinum*, indicating effects on fungal development beyond radial growth alone. Although MGI values were numerically lower in the filtrate assay than in dual culture, these values cannot be used to quantify the relative contributions of diffusible and contact-associated processes because the two assays represent different cultivation and exposure contexts. Dual culture incorporates continuous fungal growth, spatial and resource competition, hyphal contact, and ongoing production of inhibitory constituents, whereas the filtrate assay evaluates only extracellular products accumulated during prior liquid cultivation. Collectively, the time-resolved confrontation, interface morphology, and cell-free-filtrate results define a dynamic, pathogen-dependent competitive phenotype involving contact-associated and diffusible effects, while establishing clear priorities for experiments designed to resolve their respective contributions.

### 4.3 FBMN prioritizes candidate metabolites within the culture-associated chemical profile

FBMN organized the culture-associated chemical profile of *Diaporthe* sp. PBS30-7 into structurally related molecular families and highlighted features tentatively assigned to beauvericin-like cyclodepsipeptides and diketopiperazine-related metabolites. These assignments are chemically plausible within *Diaporthe*. Numerous diketopiperazines have been isolated and structurally characterized from *D. arecae* (Chang et al., 2019), and the broader occurrence of this metabolite class within the genus has been summarized by Wei et al. (2023). Beauvericin has likewise been isolated and spectroscopically confirmed from *D. Africana* (Matio Kemkuignou et al., 2023). These precedents support the occurrence of related chemical classes within *Diaporthe*. Nevertheless, because the present annotations were based on accurate mass, MS/MS fragmentation, and molecular-network relationships without authentic standards or isolated compounds, they remain putative.

Both metabolite families are biologically plausible candidates for further investigation. Beauvericins are ionophoric cyclodepsipeptides with reported antifungal activity, including effects on ion homeostasis, multidrug efflux, and fungal stress-signaling pathways (Shekhar-Guturja et al., 2016; Wang and Xu, 2012). Diketopiperazines constitute a structurally diverse class of microbial metabolites encompassing compounds with antifungal activities (Kim et al., 2023). More recently, the diketopiperazine cyclo(Pro-Tyr) was shown to damage fungal cells by disrupting plasma-membrane organization and targeting the H^+^-ATPase Pma1 (Vela-Corcia et al., 2024). These precedents provide a biological rationale for prioritizing the corresponding molecular features for experimental testing but do not establish their contribution to the antifungal phenotype of *Diaporthe* sp. PBS30-7. Unannotated features should likewise not be excluded from candidate discovery, because the absence of a library match reflects limited structural information rather than an absence of potential biological activity. Furthermore, the monoculture-derived profile may omit metabolites induced specifically during fungal confrontation. Fungal–fungal cocultivation can substantially remodel secondary-metabolite production and activate biosynthetic pathways that are silent or weakly expressed in monoculture (Wang et al., 2022).

Molecular docking provided a second prioritization layer by ranking predicted metabolite–target associations among the tentatively annotated candidates. The class- and target-dependent predictions obtained for the beauvericin analogs and cyclo(L-Tyr-L-Tyr) identified specific pairs for subsequent experimental testing. However, docking scores and static poses do not demonstrate target engagement, binding stability, or functional inhibition under biological conditions, particularly where modeled receptor structures were used. Ligand recognition may also depend strongly on receptor conformation (Wei et al., 2026). The prioritized metabolite–target pairs should therefore be regarded as testable mechanistic hypotheses rather than validated antifungal mechanisms.

### 4.4 Limitations and future perspectives

Several limitations define the scope of the present conclusions. First, chemical profiling was conducted on a pooled agar–mycelium extract from solid culture, whereas antifungal activity was evaluated using cell-free filtrates from liquid culture. These distinct cultivation systems preclude a direct association between the annotated features and filtrate activity. Moreover, profiling was limited to the ethyl acetate fraction and may have excluded highly polar, volatile, or poorly recovered bioactive constituents. Future studies should therefore apply bioassay-guided fractionation directly to active filtrates, using complementary extraction strategies across a broader polarity range. Isolation, structural elucidation, quantitative analysis, and standardized antifungal testing of individual compounds and defined combinations will be necessary to resolve their respective contributions and potential interactions.

Second, the chemical analysis was exploring. Annotations based on accurate mass, MS/MS fragmentation, and molecular-network relationships remain putative and require confirmation with authentic standards or isolated compounds characterized by orthogonal spectroscopic methods. The absence of uninoculated PDA control also prevents exclusion of medium-derived constituents and fungal transformation products. Because the pooled extract represented a single biological sample and no pooled QC sample was injected periodically, biological variability and within-run analytical drift could not be assessed. The dataset should therefore be interpreted as non-comparative candidate discovery rather than quantitative metabolomic evidence.

Third, molecular docking provided computational prioritization of candidate metabolite–target pairs but did not establish binding, target engagement, or functional inhibition (Wei C. et al., 2025). Interpretation is further constrained by putative ligand assignments and, where applicable, modeled target structures. Biochemical, cellular, and genetic validation will be required to establish causal mechanisms. Integrating these approaches with whole-genome sequencing, biosynthetic-gene-cluster analysis, and targeted metabolomics could subsequently connect biosynthetic potential with confirmed metabolite production and biological function.

## 5 Conclusion

*Diaporthe* sp. PBS30-7 exhibited a dynamic, target-dependent competitive phenotype against two *Penicillium* species, while its cell-free culture filtrate retained antifungal activity in the absence of direct fungal contact. FBMN analysis of the culture-associated extract prioritized several putative metabolite families, and molecular docking generated testable metabolite–target hypotheses; however, neither the active constituents nor their mechanisms were established. The study extends current knowledge of *Diaporthe* by documenting strain-specific environmental responses, resolving temporal and target-dependent differences in fungal confrontation, and establishing an integrated framework for prioritizing candidate metabolites without conflating chemical association with mechanistic causation. These findings provide a foundation for bioassay-guided isolation from active culture filtrates, followed by structural confirmation and functional validation.

## Data Availability

The original contributions presented in the study are publicly available. This data can be found in the MassIVE repository under accession number MSV000103237 (https://doi.org/10.25345/C5HT2GS47).
