# Supplementary material for: Competitive phenotype and metabolite repertoire of the endophytic Diaporthe sp. PBS30-7
Source: Front Microbiol. 2026 Oct 15;17:1943254. doi: 10.3389/fmicb.2026.1943254 (PMC13717619; doi:10.3389/fmicb.2026.1943254)
Supplement: Supplementary file 1 [file Supplementary_file_1.DOCX]

Supplementary Material

# 1 Supplementary Figures and Tables

## 1.1 Supplementary Figures

**Supplementary Figure 1.** Feature-based molecular network (FBMN) generated from UHPLC–Orbitrap HRMS/MS data of extracellular metabolites produced by Diaporthe sp. PBS30-7. Each node represents an individual MS/MS feature, and edges connect features with similar fragmentation spectra, indicating structural relatedness. Connected nodes form molecular families, whereas isolated nodes (singletons) represent features that did not meet the similarity threshold required for network formation. Colored nodes indicate molecular families discussed in the main text, while grey nodes represent unannotated or non-highlighted features. The complete network illustrates the overall extracellular chemical space resolved by FBMN.

**Supplementary Figure 2.** Representative base-peak chromatogram of the culture-associated extract of Diaporthe sp. PBS30-7 acquired by UHPLC–Orbitrap–HRMS in positive-ion mode. The chromatogram shows relative ion abundance across the 0–50 min analytical run.

## 1.2 Supplementary Tables

**Supplementary Table 1.** Composition of culture media used in this study

| **Medium** | **Composition(perliter)** | **Preparation Method** |
| --- | --- | --- |
| Potato Dextrose Agar | Potato starch: 4.0 g; Dextrose: 20.0 g; Agar: 15.0 g; Distilled water: 1 L | Dissolve all components in distilled water, adjust pH as required, autoclave at 121°C for 1h min. |
| Malt Extract Agar | Malt extract: 30.0 g; Enzymatic digest of soybean meal: 3.0 g; Agar: 15.0 g; Distilled water: 1 L | Dissolve all components in distilled water, adjust pH as required, autoclave at 121°C for 1h. |
| Oatmeal Agar | Oatmeal: 5.0 g; Agar: 4.0 g; Distilled water: 200 mL | 1. Weigh 5.0 g of oatmeal and place in a blender. 2. Measure 200 mL of distilled water using a 250 mL graduated cylinder. 3. Transfer both to a 500 mL bottle. 4. Heat the mixture in a water bath to extract soluble components. 5. Add 4.0 g of agar and mix thoroughly.   6. Adjust pH as required, then autoclave at 121°C for 1h. |
| pH Adjustment | 1 M HCl and 1 M NaOH (to pH 5, 6, 7, or 8) | Adjust the pH of each medium before autoclaving. |
